# The Effects of the Myobrace^®^ System on Peripheral Blood Oxygen Saturation (SpO_2_) in Patients with Mixed Dentition with Oral Dysfunction

**DOI:** 10.3390/dj11080191

**Published:** 2023-08-09

**Authors:** Luca Levrini, Roberta Persano, Sofia Piantanida, Andrea Carganico, Alessandro Deppieri, Giulia Naboni, Rodolfo Francesco Mastrapasqua, Stefano Saran

**Affiliations:** 1 Department of Human Sciences, Innovation and Territory, School of Dentistry, Postgraduate of Orthodontics, University of Insubria, 21100 Varese, Italy; rpersano@studenti.uninsubria.it (R.P.); spiantanida1@studenti.uninsubria.it (S.P.); acarganico@studenti.uninsubria.it (A.C.); adeppieri@studenti.uninsubria.it (A.D.); gnaboni@studenti.uninsubria.it (G.N.); ssaran@studenti.uninsubria.it (S.S.); 2 ENT Department, Rivoli Hospital, ASL to 3, 10098 Torino, Italy; rfmastrapasqua@aslto3.piemonte.it

**Keywords:** mixed dentition, malocclusion, myofunctional therapy, oxygen saturation, orthodontics

## Abstract

Introduction: Myobrace^®^ is an orthodontic device that has the purpose of correcting oral dysfunctions, thus predisposing the physiological growth of the jaws, aligning teeth, and optimizing face development. This device is usually associated with Myobrace^®^ Activities to reach this target. Considering the lack of studies in the literature about peripheral blood oxygen saturation (SpO_2_) and the use of preformed oral devices, the aim of this study is to quantify the change in blood oxygen saturation (SpO_2_) in patients treated with the Myobrace^®^ System in mixed dentition. Materials and Methods: In this study, 23 children (11 females and 12 males) were involved, who were affected by different oral dysfunctions and were treated with a Myobrace^®^. Blood oxygen saturation measurements were taken at baseline and after every four months for a year. The SpO_2_ measurements were taken in the rest position and with a closed mouth for a total of 12 min—6 min with and 6 min without the Myobrace^®^ oral device. All data points were anonymized and recorded on an Excel spreadsheet. A statistical analysis was carried out. Results: Therapy with a Myobrace^®^ in patients with mixed dentition resulted in a statistically significant increase in oxygen saturation. In particular, in patients with a closed mouth, a statistically significant increase in oxygen saturation was observed, bringing it from 97.66% to 99.00%, while in the rest position, the increase was from 98.03% to 99.07%. Conclusions: The use of Myobrace^®^ devices in patients with mixed dentition could lead to a significant improvement in blood oxygen saturation.

## 1. Introduction

Several studies have highlighted the relationship between dysfunctional oral habits and malocclusions [1,2,3]. In fact, incorrect myofunctional habits like oral breathing, thumb sucking, and tongue thrust swallowing may have a negative impact on dental and maxillofacial development during childhood.

Mouth breathing is one of the most frequent oral dysfunction in young patients, with a prevalence ranging from 11% to 56% [4], and it is also an important symptom of sleep disorders. It is considered a pathological condition when 80% of the inhaled air passes through the mouth rather than through the nose.

Mouth breathing is a symptom representing various degrees of obstruction of the upper airways, due to different causes like hypertrophy of the tonsils and adenoids, a deviated nasal septum, a reduction in the nasopharyngeal space, nasal polyposis, nasofacial trauma, allergic rhinitis, chronic sinusitis, nonspecific infections, and all the conditions that reduce the nasal airflow, forcing the air to enter completely or partially through the oral cavity. If the upper-airway obstruction is not removed, the child is forced to breathe through the mouth, over time causing changes to the physiological craniofacial growth and to the patient’s general health [4]. The effects of mouth breathing on facial growth and dental occlusion have been identified in various articles [5,6,7]. Already in 1981, Harvold et al. [5] led an experiment on rhesus monkeys in which they found that rhesus monkeys with an artificial nasal obstruction maintained a lower position of the mandible, developing a steeper mandibular plane and an increase in facial height compared to control animals. The same happens in children with mouth breathing who often present a long face, maxillary protrusion, mandibular retrusion, and posterior rotation. 

For this reason, early treatment of this dysfunction is essential.

Correct breathing is also necessary to ensure restful sleep. Often, sleep is altered by respiratory disorders or by actual episodes of nocturnal apnea caused by soft tissue friction or contact applied by gravity and changes in muscle tone during sleep.

In fact, children who breathe through their mouths may develop episodes of apnea or hypopnea during sleep time, developing various sleep disorders: OSAS (obstructive sleep apnea syndrome), SIDS (Sudden Infant Death Syndrome), ALTEs (Apparent Life-Threatening Events), and UARS (Upper Airway Resistance Syndrome). Mouth breathing is a sign of varying degrees of upper airway obstruction brought on by a variety of conditions, including allergic rhinitis, chronic sinusitis, nonspecific infections, tonsil and adenoid hypertrophy, a deviated nasal septum, reduced nasopharyngeal space, nasal polyposis, nasofacial trauma, and nasal polyposis [8,9].

Obstructive sleep apnea syndrome (OSAS) is a condition caused by a complete or partial obstruction of the upper airways that occurs during sleep, characterized by prolonged episodes of interruption (apnea) or a decrease (hypopnea) in the oral airflow, resulting in decreased blood oxygen saturation.

The immediate consequence is disturbed sleep, characterized by snoring, deriving from inspiratory effort, and micro-awakenings or real awakenings of the patient in an attempt to restore the air flow. The fragmentation of sleep, in turn, results in a series of disturbances in the waking state, such as morning headaches, drowsiness, irritability, and difficulty concentrating [10].

From the literature, it emerges that the prevalence of OSAS in the general population is approximately 13% for men and 6% for women, with a prevalence in the general adult population ranging between 9% and 38% [11]. However, it can affect 2–5% of children of all ages and is most common in children between 2 and 6 years of age.

At the pathophysiological level, OSAS is based on a partial or complete obstruction of the airways during sleep, which derives from a collapse of the tissues that form the PAS (posterior airway space). This collapse can occur at different points: affecting the soft palate (the most common), from the walls of the pharynx, the base of the tongue, or the epiglottis [12].

An essential tool for diagnosis is polysomnography, a specialist examination that, during sleep time, evaluates various cardiorespiratory parameters like sleep “architecture”, endopharyngeal pressure, thoracoabdominal movements, nasal flow, heart rate, oxygen saturation, and snoring. The final data from the detection of all these parameters are the apnea/hypopnea index, or AHI, which is the number of apneas per hour.

The value of AHI allows you to classify the OSAS according to the severity: up to five events an hour is considered a normal value, while it is pathological to have more than five apnea events per hour. In particular, a range from 5 to 15 apnea events defines mild OSAS; from 15 to 30, it is moderate OSAS; and over 30 events defines severe OSAS.

Therapies for OSAS depend on the gravity of the condition. For mild-to-moderate OSAS, oral devices can represent a solution. These oral appliances, to be worn during sleep, increase the upper-airway space through a mechanical action via two different strategies: to lead the tongue into a more advanced position (tongue-retaining devices) and to determine mandibular advancement (mandibular advancement device or MAD). These devices are well tolerated by patients, easy to use, noninvasive, and removable, with mild and, overall, acceptable side effects [12].

### Myofunctional Therapy in Breathing Disorders

Breathing disorders and OSAS can also be prevented by myofunctional therapy, neuromuscular toning, and a re-education of the oral, facial, and pharyngeal muscles (lips, tongue, cheeks, face, and throat) through a series of activities that help to normalize the development and function of the head–neck district and the overall blood oxygen saturation. 

Myofunctional therapy is based on oral exercises and oral appliances that have the purpose of training the neuromuscular biological structures. It uses the forces that are inside the oral district to influence dental movement and the growth of the mandible. 

More specifically, myofunctional therapy is an educational and rehabilitative program which uses the application of muscular forces through the use of therapeutic devices and exercises, with the aim of correcting dysfunctions that affect the orofacial system, such as dysfunctional swallowing, oral breathing, alterations in muscle tone, alterations in chewing, occlusion, voice and speech articulation, as well as problems affecting the temporomandibular joint or oral dysfunctions such as prolonged thumb sucking [13].

Among these therapeutic devices, there is the Myobrace^®^, a prefabricated orthodontic device that aims to prevent and correct orofacial dysfunctions, predisposing the physiological growth and development of the jaws to align teeth, to optimize face development, to promote the transition from oral to nasal breathing, and to correct dysfunctional swallowing [14,15].

However, for a successful outcome, it is essential that the use of these oral appliances is associated with an educational program, known as Myobrace^®^ Activities, which consists of a series of exercises with the aim of promoting nasal breathing, swallowing and the correct position of the tongue and the cheeks, and to further correct oral dysfunction that is impacting dental and jaw development. The educational activities must be performed twice a day, in conjunction with using the Myobrace^®^ device.

No studies in the literature have investigated the effects of preformed devices on peripheral blood oxygen saturation and, thereby, their ability to improve respiration effectiveness. This study aims to evaluate objective improvements in the respiratory function of patients with oral or mixed respiration through oximetry values after the application of a Myobrace^®^ and the related activities. The null hypothesis assumes that the Myobrace^®^ system does not affect peripheral blood oxygen saturation in patients with mixed dentition with oral dysfunction. The working hypothesis is that the use of this device improves SpO_2_.

## 2. Materials and Methods

Twenty-three (23) patients (11 females and 12 males) affected by different oral dysfunctions treated with a Myobrace^®^ system were enrolled in the study between September 2021 and January 2023. 

The selected group included patients aged from 6 to 13 years old in mixed dentition treated at the dental department of the “Ospedale di Circolo, fondazione Macchi”, University of Insubria, Varese, Italy.

The parents of the patients signed an informed consent form and no financial incentive was given for participation. The research was approved by the ethics committee n. 0111335 of “Università degli Studi dell’Insubria”, Varese, Como, Italy. 

The inclusion criteria of the group were as follows: using Myobrace^®^ therapy during the observation period, completed at least two out of three stages of the Myobrace^®^ Activities, presented with a breathing disorder, mixed dentition, and the sum of decayed, missing, having caries, and filled teeth (dmft index) being under 4. 

Those patients who underwent only one out of three stages of therapy were not included.

After the first measurement (baseline), the ones with an initial oxygen saturation (SpO_2_) without the oral device over or equal to 99% were excluded because they did not qualify as having a breathing disorder.

Every 4 months for a total of 12 months, each enrolled patient was monitored with a Rad-G pulse oximeter to evaluate the oxygen blood saturation with and without a Myobrace^®^. A blood oxygen saturation measurement was taken at baseline with a closed mouth and in a rest position using a K-series Myobrace^®^. The rest position was the physiological spontaneous posture of the lips, which can be different in relation to the patients’ breathing dysfunction. 

For all patients, the total observation time was 12 min, of which 6 min was with the preformed device and 6 min was without.

The measurements were taken every 4 months with the different devices that were needed as the patient grew, starting from device 1 (K1), then in the following 4 months with device 2 (K2), and at the end of the 8th month with device 3 (K3). 

For each patient, the maximum, minimum, and mean SpO_2_ values were recorded.

The reading values of the oxygen saturation are marked in Table 1.

### Device Description

The Myobrace^®^ is an orthodontic device with an interceptive function. It has the aim of re-educating the oral muscles to correct oral dysfunction, predisposing the physiological growing of the jaws, aligning teeth, and optimizing face development. For this reason, it comes under myofunctional therapy. 

The Myobrace^®^ System consists of a series of intraoral appliances that are worn for one hour a day as well as during the night (during sleep). 

The Myobrace^®^ is a prefabricated removable device in silicone which is characterized by the presence of a buccal and lingual shield, a tongue tag, and a tongue elevator.

The buccal shield discourages the hyperactivity of the orbicularis and buccinator muscles and, therefore, the centripetal force exerted by the vestibular muscles; the tongue tag stimulates the tip of the tongue to find its correct position in contact with the palatine wrinkles, behind the upper incisors, indirectly facilitating the correct lip seal and physiological nasal breathing; and the tongue elevator favors the lifting of the tongue. Furthermore, according to the recent literature, a Myobrace^®^ System can move the mandible forward with a possible increased of the posterior airway space (PAS) [14].

A single device also has different characteristics depending on the model used and, therefore, on the treatment stage.

In fact, the Myobrace^®^ System offers 5 different programs depending on the dentition and age of the patient, which involve the use of 3–4 devices to be replaced based on the progression of orthodontic therapy. We can distinguish between them as follows:

1. The J (junior) series: Used during the primary dentition, and therefore approximately between 2 and 5 years of age. This device corrects oral dysfunctions by promoting correct chewing and nasal breathing.
2. The K (kids) series: Used in the mixed-dentition phase, approximately between 6 and 10 years of age. It is particularly indicated in cases of dental crowding, for the correction of open bites and deep bites due to oral dysfunction habits.
3. The T Series, for teens: Used during the development phase of permanent teeth, roughly between 11 and 15 years of age. Its objective is to promote the correct development of the dental arches and the alignment of the teeth.
4. A series, for adults: Used after age 16 and for adults. This type of device is indicated to correct many types of dental malocclusions and related problems such as TMJD, joint clicks, speech problems, or snoring.
5. Series I-3: used in the early mixed-dentition stage to intercept class-III dental malocclusions.

In this study involving children in mixed dentition, they were all treated with K-series devices [15,16].

## 3. Results

The obtained results report a change in the oxygen saturation while using the Myobrace^®^ device.

### Statistical Analysis

The oxygen saturation for every measure was reported. All data points were recorded and anonymized on an Excel spreadsheet (Microsoft Corporation, Redmond, WA, USA (2018). *Microsoft Excel*). The data were analyzed using SPSS 25 (IBM Corp. (2017), IBM SPSS Statistics for Windows. Armonk, NY, USA, IBM Corp.).

Saturation levels were tested for normality with the Kolmogorov–Smirnov test and turned out as non-normally distributed. Data points were confronted with the Wilcoxon signed-rank test for paired samples and expressed as a median (interquartile range) (Table 2).

Significance was set as *p* < 0.05 (Table 3). A sample size of 24 subjects was required to detect an effect size of 0.06 (statistical power 0.8). A single dropout was registered due to scheduling conflicts. 

Table 1 reports the patients of the sample, indicating the type of device used (K1-K2-K3) and the SpO_2_ detected in the different positions. 

Table 4 shows that the mean percentage of SpO_2_ without a Myobrace^®^ in the rest position was 98.03%.

The mean percentage of SpO_2_ with a Myobrace^®^ in the rest position was 99.07%. 

There was an improvement of 1.04 points, which is statistically significant.

The mean percentage of SpO_2_ without a Myobrace^®^ with a closed mouth was 97.66%, while the mean percentage of SpO_2_ with a Myobrace^®^ with a closed mouth was 99.00%. It can be observed that there was an increase in oxygen saturation of 1.34 points due to the use of the device with a closed mouth.

According to the data, there was a statistically significant improvement in oxygen level in the patients using a Myobrace^®^, both with a closed mouth and with a mouth at rest.

The use of the Myobrace^®^ resulted in a higher increase in oxygen saturation with the device in the mouth than without. In particular, the blood oxygen level was higher when patients used the oral devices with a closed mouth rather than patients at rest without a device, suggesting that the Myobrace^®^ determined an increase in oxygen saturation.

## 4. Discussion

According to the results, it is possible to affirm that the use of a Myobrace^®^ can determine an increase in the posterior–anterior space (PAS) due to the forward position of the mandible, which improves the blood oxygen saturation.

The ability of the Myobrace^®^ to change the posture of the mandible has been reported in the literature; Usumez and collaborators [14] demonstrated that the Myobrace^®^, if worn overnight for 10 to 12 h, is able to move the mandible forward thanks to acting on the orofacial muscles.

In this specific case, the Myobrace^®^ stretches the muscles of the oral complex (the superficial masseter, medial pterygoid, and inferior head of the lateral pterygoid) that protrude the mandible. The stretching of muscles during the night causes the production of lactic acid and produces muscular fatigue [17,18]. In the morning, when the Myobrace^®^ is removed from the mouth, the muscles react by increasing the frequency of contractions to reduce the lactic acid [19]. The hypercontractility determines a higher level of oxygen in the muscles. This permits the undifferentiated cells to evolve in myoblasts, increasing the number of muscular fibers and, therefore, the muscles’ mass [20]. Since these specific muscles are therefore stronger, they are able to keep the mandible in the new, more protruded position. This biological aspect determines the new position of the jaw which is protruded. Moreover, the mandibular advancement allows a protrusion of the tongue base and, therefore, an enlargement of the posterior air space.

The PAS is normally reduced in children who breathe through the mouth instead of the nose [21]. This is due to different causes, like the hypertrophy of tonsils and adenoids, which is the main cause of OSAS in children between the ages of 3 and 6 years, the period of greatest development of lymphoid tissue [22]; a retruded mandible relative to the base of the cranium and the maxilla, due to the low position of the jaw and tongue during breathing; and the retropositioning of the tongue. 

Other causes of oral breathing that determine the reduction in the posterior airspace can be a deviated nasal septum, nasal polyposis, nasofacial trauma, allergic rhinitis, chronic sinusitis, and nonspecific infections.

If these causes are not removed in a growing child, they can negatively alter craniofacial development. In fact, an oral respiratory model determines changes in the lingual and lip posture, which, in turn, determine skeletal alterations due to the action of the neuromuscular system. There is a biological theory according to which the growth of the jaws is directed and accelerated by thrust and muscular balance phenomena. Normally, the thrust exerted by the tongue is directed toward the palate, while the cheeks and lips exert pressure in a centripetal direction on the jaw, allowing for balanced growth [23].

In children with oral breathing, there is an imbalance in this muscular system: the tongue remains in an intermediate or low position and tends to get between the lips, altering the position of the teeth and the swallowing function [24,25].

Due to the lack of pressure of the tongue against the palate, determinate by its lower position, there is an unsuccessful development of the palate in the transverse direction and a contraction of the upper arch [26]. A low lingual position is also associated with a hyperdivergent growth pattern and mandibular retrusion [27].

The thrust of the tongue on the anterior elements causes a vestibularization of the upper incisors, which can be associated with an anterior open bite and a class-II malocclusion.

For all these reasons, it is essential to correct the oral breathing pattern by promoting the nasal one. This is possible through the use of the Myobrace^®^ System and Activities (exercises) that influence the PAS.

The variation in the PAS produced by the use of a Myobrace^®^ could be comparable to the one obtained with the use of the MAD in the treatment of OSAS. The Myobrace^®^ is able to increase the airway’s caliber and avoids its collapse because of the negative pressure that develops during inspiration. A cephalometric analysis of patients treated with MAD demonstrated an increase in the PAS. Moreover, the mandibular advance device activates the neuromuscular system of the airways that increases muscle tone, which in turn prevents the walls from collapsing and keeps the upper airways open [10].

Consequently, the enlargement of the PAS allows obtaining an increase in the airflow, in particular of oxygen. Once the O_2_ molecules reach the pulmonary alveoli, they transfer to the blood circulation in the pulmonary capillaries, thus increasing the partial pressure of oxygen [28]. Our results show an increase in blood oxygen saturation after using the Myobrace^®^ all night long plus one hour a day for approximately 12 months. The obtained results are very encouraging considering the affordable cost of this kind of device and the very important benefits the patients can have.

## 5. Limitations

The study has limitations caused by the complexities of the oral dysfunction. The most important limitation is the size and the selection of the sample considered. An issue could be the possibility of low compliance of the patients in using the devices and the impossibility of knowing the quality and the quantity of oral exercises practiced. 

## 6. Conclusions

The results of the study suggest that the use of a Myobrace^®^ improves blood oxygen saturation. The benefits could be related to the following:

- The mandibular advancement caused by the device, with an immediate increase in the upper airway.
- An increase in the PAS that is comparable to the effects of the MAD.

It will be necessary to proceed with the collection of data on a larger sample of patients to confirm these results and other possible benefits over a longer period of time.

## Figures and Tables

**Table 1:** The type of device used (K1-K2-K3) and the SpO_2_ detected in the different positions.

		OXYGEN SATURATION			
		Without Myobrace Mouth at Rest	Without Myobrace Closed Mouth	With Myobrace Mouth at Rest	With Myobrace Closed Mouth
**Patient 1**	K1	97	96	99	99
	K2	98	97	100	100
	K3	98	98	100	100
**Patient 2**	K1	98	98	99	99
	K2	98	98	99	100
	K3	100	99	100	100
**Patient 3**	K1	96	95	98	98
	K2	96	96	100	99
	K3	97	97	100	100
**Patient 4**	K1	98	98	100	100
	K2	99	98	100	100
	K3	100	99	100	100
**Patient 5**	K1	98	98	99	99
	K2	98	99	100	100
**Patient 6**	K1	98	97	100	99
	K2	98	98	99	99
	K3	100	99	100	100
**Patient 7**	K1	98	98	99	100
	K2	100	100	99	100
	K3	100	99	100	100
**Patient 8**	K1	98	98	99	99
	K2	99	99	100	99
	K3	100	100	100	100
**Patient 9**	K1	97	97	98	99
	K2	98	97	99	99
	K3	99	99	100	100
**Patient 10**	K1	96	95	96	97
	K2	97	97	98	98
	K3	98	97	99	99
**Patient 11**	K1	97	97	98	98
	K2	98	97	98	98
**Patient 12**	K1	98	98	99	98
	K2	99	99	100	100
	K3	99	98	100	99
**Patient 13**	K1	95	95	96	96
	K2	97	97	98	99
	K3	98	98	99	100
**Patient 14**	K1	98	98	100	99
	K2	99	99	100	100
	K3	100	99	100	100
**Patient 15**	K1	96	95	97	96
	K2	97	97	99	99
	K3	98	98	100	100
**Patient 16**	K1	96	96	97	96
	K2	97	97	99	98
**Patient 17**	K1	98	98	100	99
	K2	99	99	100	99
	K3	100	100	100	100
**Patient 18**	K1	97	97	99	98
	K2	99	98	100	98
**Patient 19**	K1	98	98	100	100
	K2	100	99	100	100
**Patient 20**	K1	97	97	99	99
	K2	99	97	100	99
	K3	98	98	100	100
**Patient 21**	K1	96	95	96	96
	K2	97	97	99	99
	K3	98	97	100	100
**Patient 22**	K1	98	98	100	99
	K2	100	100	100	99
**Patient 23**	K1	97	96	98	97
	K2	98	97	98	98

**Table 2:** The Wilcoxon signed-rank test for paired samples and expressed as median (interquartile range).

	Median of the Oximeter Values
**Baseline open mouth**	98 (2)
**Baseline closed mouth**	98 (2)
**Device open mouth**	100 (1)
**Device closed**	99 (1.25)

**Table 3:** Level of significance.

	With Myobrace vs. without Myobrace Significance
**Open mouth**	*p* < 0.01
**Closed mouth**	*p* < 0.01

**Table 4:** The mean percentage of SpO_2_.

	Mean of Oxygen Saturation Values
**Without Myobrace mouth at rest**	98.03
**Without Myobrace closed mouth**	97.66
**With Myobrace mouth at rest**	99.07
**With Myobrace closed mouth**	99.00

## Data Availability

Data are available upon request from the corresponding authors.
